# Case of T-B+NK+ X-Linked Severe Combined Immunodeficiency Disease

**DOI:** 10.1155/2024/4278595

**Published:** 2024-10-17

**Authors:** Wenya Qian, Min Wu, Guanling Wang

**Affiliations:** Department of Pediatrics, Women and Children's Hospital of Ningbo University, Ningbo, Zhejiang, China

## Abstract

We report a case of T-B+NK+ severe combined immunodeficiency disease (SCID) caused by *IL2RG* gene mutation (NM_000206.3 [*IL2RG*]: c.925-2A > G). The patient, a 2-month-old male, experienced multiple infections and decreased white blood cells in the early postnatal period. Antibiotic treatment was ineffective and ultimately resulted in multiple organ failure. The second-generation gene sequencing of patient showed that the *IL2RG* gene had a hemizygous mutation NM_000206.3 (*IL2RG*): c.925-2A > G, indicating a classical splice site mutation. According to the guidelines of the American College of Medical Genetics (ACMG), NM_00206.3 (*IL2RG*): c.925-2A > G variants can be classified as pathogenic (PVS1&PM1&PM6).

## 1. Introduction

Severe combined immunodeficiency (SCID) is a rare and deadly complex genetic disease with diverse causes, characterized by a common loss of T and B lymphocyte function, including natural killer (NK) lymphocyte function in some cases [1]. Its incidence rate is estimated at 1/50,000 to 1/100,000 births [2]. Patients with SCID are usually newborns or infants, who often exhibit abnormalities in T-cell count, function, and serum antibody parameters and can be associated or not with B-cell and/or NK-cell lymphopenia. They are prone to recurrent infections of various pathogens such as viruses, bacteria, and fungi, as well as immune-related diseases such as malignant tumors and autoimmune diseases [3]. The general treatment principle of SCID is to prevent and control infection, while hematopoietic stem cell transplantation and gene therapy are currently the best treatment options for applicable patients [4]. Here, we report a case of T-B+NK+ X-linked SCID (X-SCID) caused by a rare gene mutation.

## 2. Case Presentation

A 2-month-old male presented to our hospital due to recurrent diarrhea after birth and poor feeding. He was a full-term baby born naturally, the first child of his parents, with a birth weight of 2.4 kg. The parents were in good health and denied intermarrying, while the mother had not been exposed to toxic substances or radiation during pregnancy. There was no indication that his relatives had immune deficiencies or genetic diseases.

After admission, the physical examination showed that the child was mentally depressed, malnourished, and physically weak, with an enlarged liver (5 cm below the ribs) and active bowel sounds (6–8 times per minute). Immunological evaluation revealed panhypogammaglobulinemia. Lymphocyte subpopulation analysis indicated: total white blood cell count 2.4 × 10^9^/L (normal: 5.0–12.0 × 10^9^/L), total CD3^+^ T cells 4.7% (normal: 53.0%–75.0%), CD3^+^CD4^+^ T cells 0.8% (normal: 29.0%–53.0%), CD3^+^CD8^+^T cells 3.3% (normal: 13.5%–27.0%), CD16^+^CD56^+^ NK cells 17.5% (normal: 7.0%–19.0%), total CD19^+^ B cells 73.7% (normal: 2.0%–30.0%), CD4^+^/CD8^+^ 0.2 (normal: 1.1–23.9). The results showed a significant decrease in the proportion of CD3, CD4, and CD8 cells, with an inverted CD4/CD8 ratio, while the proportion of NK cells was normal and the proportion of B cells increased. In addition, the results of immunoglobulin assay showed: IgG 2.7 g/L (normal: 3.1–15.6 g/L), IgA 0.07 g/L (normal: 0.3–2.6 g/L), and IgM 0.08 g/L (normal: 0.5–2.0 g/L) (Table 1). Pathogenic examination suggests the presence of multiple infections such as cytomegalovirus, *Klebsiella pneumoniae*, *Escherichia coli*, and smooth Candida. Furthermore, cardiac ultrasound detected patent foramen ovale.

During hospitalization, supportive treatment such as anti-infection, correction of anemia, and fluid replacement was given. However, the patient's condition continued to progress, with multiple organ dysfunctions such as respiratory difficulties, pulmonary bleeding, upper gastrointestinal bleeding, liver function damage, electrolyte disorders, and glucose metabolism disorders. Blood culture suggests *Staphylococcus aureus* infection. After 10 days of admission, the patient was transferred to pediatric intensive care unit for further treatment, assisted by ventilation. However, on the 12th day of hospitalization, the infant suddenly experienced a decrease in oxygen saturation, a decrease in heart rate, and bloody fluid from endotracheal intubation. We have provided rescue measures such as cardiopulmonary resuscitation, adrenaline injection, and the use of vasoactive drugs, but the condition of the patient did not improve. His family ultimately decided to give up treatment and request discharge.

**Figure 1:**
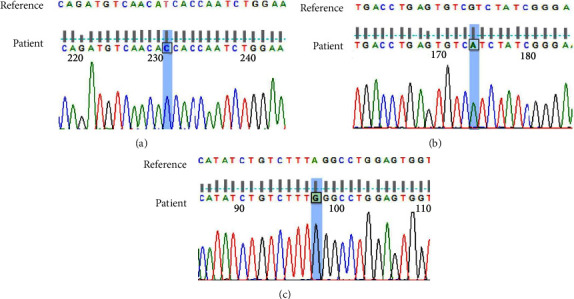
Gene sequencing map of the patient. (a) *IL7R* gene exon 2 c.197T > C homozygous missense mutation. (b) *IL7R* gene exon 4 c.412G > A homozygous missense mutation. (c) *IL2RG* gene intron 7 c.925-2A > G intron hemizygous mutation.

Due to significant abnormalities in the baby's blood routine and lymphocyte subsets, we collected the baby's peripheral blood for genetic testing. The results showed that there was a missense mutation of c.197T > C (p.Ile66Thr) in exon 2 and a missense mutation of c.412G > A (p.Val138Ile) in exon 4 of the *IL7R* gene (NM_002185.5), both of which were classified as benign (BA1) based on the American College of Medical Genetics (ACMG) guidelines. However, there was a hemizygous mutation in the intron 7 of the *IL2RG* gene (NM_000206.3), with mutation sites c.925-2A > G (Figure 1); based on the ACMG guidelines, this variant can be classified as pathogenic (PVS1 &PM1&PM6). Finally, the patient was diagnosed with X-SCID.

## 3. Discussion

SCID is the most severe type of primary immunodeficiency disease, which is associated with multiple gene mutations. According to the 2022 update on the classification from the International Union of Immunological Societies (IUIS) expert committee, there are at least 19 genes related to the occurrence of SCID, corresponding to different lymphatic phenotypes (Table 2) [5]. Moreover, the number of SCID-causing genes is still increasing with novel gene discoveries. For instance, POLD3 deficiency has been recently reported as a novel genetic cause of severe syndromic SCID [6]. X-SCID is the most common type of SCID, accounting for approximately 50% of patients with SCID. It is an X-linked recessive inherited immunodeficiency disease; only males have this type of SCID, but females may carry the gene and have a 1 in 2 chance (50%) of passing it on to each son [7].

In 1993, Noguchi et al. [8] and Puck et al. [9] localized the X-SCID pathogenic gene to *IL2RG* on the X chromosome q13.1, which has a total length of 4705 bases and eight exons and can encode IL-2R*γ* chain protein containing 369 amino acids. IL-2R*γ* chains are functional units shared by various cytokine receptors such as IL-2, IL-4, IL-7, IL-9, IL-15, and IL-21 and are signaling transduction molecules that regulate the differentiation, development, and maturation processes of T cells, NK cells, and B cells [10]. The integrity of the IL-2R*γ* chain is crucial for the body's immune function [10, 11]. IL-2R*γ* chain dysfunction can lead to impaired intracellular signaling during T-cell development, causing T-cell development to stagnate in the pre–T-cell stage and resulting in severe T-cell defects. T-cell function plays an important role in the maturation of B cells; therefore, T-cell defects also lead to B-cell dysfunction [8]. IL-15 is involved in regulating the development of NK cells, and immune system abnormalities in patients with X-SCID typically manifest as a significant decrease in T cells and NK cells. The number of B cells is normal or increased, but their function is abnormal, leading to a decrease in immunoglobulin production and class switching disorders [12].

As of 2023, the Human Gene Mutation Database (HGMD) database includes a total of 278 *IL2RG* gene mutations (HGMD Professional, April 2023). The most common mutation in *IL2RG* is a missense or nonsense mutation caused by a single base change, and a total of 144 types have been found so far. The second common is splicing mutations, with approximately 42 types. In this case report, the patient suffered a c.925-2A > G (Figure 1) mutation in the seventh intron region of *IL2RG* gene (NM_000206.3), which belongs to the classical cleavage site. We searched databases such as PubMed, ClinVar, UniProt Humsavar, dbSNP, Ensembl, and HGMDpro and found no reports of this mutation. In order to investigate this point mutation without clear pathogenic reports, we further referred to the Gene Mutation Interpretation Guidelines of ACMG and found that the site conforms to the pathogenic mutation (PVS1), no population carrying rate (PM2), and no newly discovered mutation verified by parental samples (PM6). The final grading was determined to be pathogenic. The main clinical symptoms of patient in this case are recurrent diarrhea, malnutrition, multiple infections, drug-resistant bacterial infections, significantly a lower T cell ratio, and a correspondingly higher B-cell ratio. However, various immunoglobulins have decreased, indicating abnormal B-cell function. Such severe immune dysfunction is consistent with the characteristics of immunodeficiency disease. Combined with the results of genetic testing, the case was ultimately determined to be X-SCID caused by *IL2RG* mutation.

On the other hand, the typical manifestation of X-SCID caused by *IL2RG* mutation is T-B+NK-, which is inconsistent with the T-B+NK+ manifestation in this case. According to a literature review, although T cell and NK cell deletions coexist in most *IL2RG* variant severe combined immunodeficiency children, there are also a small number of cases with normal NK cell counts [13, 14]. Due to the fact that *γ*-chain is an important component of IL-15 and IL-7 expression, and IL-15 expression defects can cause NK-, the presence of a T-B+NK+ phenotype may be caused by mutations that lead to the preferential preservation of IL-15R-mediated signalling [13]. Another report also provides a similar explanation, where point mutations cause an amino acid substitution in the extracellular domain of *γ*chain, leading to selective defects of IL-4R and IL-7R-mediated signalling with relative preservation of signalling through the IL-2R and IL-15R [14]. In addition, we further searched for literature reporting mutations near c.925 and found that c.924+1G > A and c.924G > A are both pathogenic mutations of *IL2RG*. Interestingly, the c.924G > A mutation is also a splicing mutation and may manifested as T-B+NK+ [15]. Yamashita et al. found that c.924 is the last nucleotide of the exon 7, and its mutation can cause splicing abnormalities (including skipping exon 7 splicing or splicing in more regions), but still retains some normal splicing. The different proportions of aberrant splicing and leaky production of normally spliced *IL2RG* mRNA may result in different clinical manifestations and immune phenotypes. Due to the presence of normal splicing, some of the normal expression of gamma chains is retained, resulting in a normal or mild to moderate decrease in the number of NK cells but a decrease in their response to cytokine stimulation [15]. In our case, c.925–2 is the second to last nucleotide of intron 7, and its mutation may produce similar effects to the c.924 mutation, namely, abnormal splicing of mRNA in the same region and similar immune phenotypes. Therefore, the reason for the T-B+NK+ phenotype in this case may be consistent with the mechanism explained in Yamashita et al.'s study.

There are also two mutation sites in the exon 2 and exon 4 of the *IL7RG* (NM_002185.5) in this case. Anne Puel et al. have reported these two mutation sites, suggesting that they are associated with a case of SCID caused by an *IL7R* mutation [16]. However, in the Exome Sequencing Project, 1000 Genomes Project, and Exome Aggregation Consortium Project, the mutation frequency of these two loci is greater than 5%, and according to the ACMG guideline, they are classified as BA1. Therefore, in this study, these two mutations were ultimately considered nonpathogenic. Unfortunately, we did not conduct further laboratory genomic analysis on the case to validate this conclusion.

General treatment principle of SCID is to prevent and control infection, mainly including avoiding live vaccines, strict isolation, supplementing immunoglobulins, and applying antibiotics [17]. The curative measure for X-SCID is to rebuild the patient's immune system, mainly including hematopoietic stem cell transplantation (HSCT) and gene therapy [18]. Stem cell transplantation can choose from bone marrow and umbilical cord blood stem cells or peripheral blood transplantation. Compared to HSCT, the technology of gene therapy for immunodeficiency is not yet mature. On the one hand, it may lead to adverse outcomes such as malignant tumors, and on the other hand, in current gene therapy protocols, achieving full correction of the genetic defect is not consistently attractive [4]. In addition, previous literature has shown that age is an important factor affecting the transplant survival rate of patients with X-SCID. If HSCT is performed on patients within 3.5 months after birth, the transplantation survival rate can reach 94%, and the survival rate decreases with increasing age and infection opportunities [17]. Therefore, early *IL2RG* gene testing is of great significance for the diagnosis and treatment prognosis of patients with X-SCID.

The T-cell receptor excision circle (TREC) assay is an effective screening tool for SCID. It can help us screen for SICD patients in the neonatal period, providing more time for subsequent treatment [19]. Unfortunately, the patient in this report experienced recurrent diarrhea and malnutrition shortly after birth. Although SCID was diagnosed during this admission, severe multiple infections had already occurred, and there was no opportunity for bone marrow transplantation. Eventually, the patient died due to multiple organ failure.

## 4. Conclusion

The presented case was a T-B+NK+X-SCID caused by a rare gene mutation, which reveals the clinical manifestations and diversity of gene mutations in SCID and highlights the importance of genetic testing for diagnosis and treatment. With the widespread promotion of TREC in clinical practice and the continuous development of technologies such as bone marrow transplantation and gene therapy, early diagnosis and treatment may provide more opportunities for survival in children with SCID.

## Figures and Tables

**Table 1:** Laboratory data and immunological characteristics of patients.

**Test**	**Patient**	**Normal value**
White blood cell count (× 10^9^/L)	2.4	5.0–12.0
Absolute neutrophil count (× 10^9^/L)	0.5	1.8–6.3
Absolute lymphocyte count (× 10^9^/L)	1.5	1.1–3.2
Hb (g/L)	9.7	11.0–12.0
PLT (× 10^9^/L)	101	125–350
IgG (g/L)	2.7	3.1–15.6
IgA (g/L)	0.07	0.3–2.6
IgM (g/L)	0.08	0.5–2.0
Total CD3^+^ T cells (%)	4.7	53.0–75.0
CD3^+^CD4^+^ T cells (%)	0.8	29.0–53.0
CD3^+^CD8^+^ T cells (%)	3.3	13.5–27.0
CD16^+^CD56^+^ NK cells (%)	17.5	7.0–19.0
Total CD19^+^ B cells (%)	73.7	12.0–30.0
CD4^+^/CD8^+^	0.2	1.1–23.9

**Table 2:** Immunophenotype of SCID.

**Phenotype**	**Defective gene**	**Type of disease**	**Hereditary**
T-B+NK-	*IL2RG*	X-linked SCID	X-linked
	*JAK3*	JAK3 deficiency SCID	Autosomal recessive

T-B+NK+	*IL7RA*	IL7R deficiency SCID	Autosomal recessive
	*CD3D CD3E CD3Z*	CD3 complex component deficiency SCID	Autosomal recessive
	*PTPRC*	CD45 deficiency SCID	Autosomal recessive
	*CORO1A*	Coronin-1A deficiency SCID	Autosomal recessive
	*LAT*	LAT deficiency SCID	Autosomal recessive
	*LCP2*	SLP76 deficiency	Autosomal recessive

T-B-NK+	*RAG1 RAG2*	Omenn syndrome	Autosomal recessive
	*LIG4*	DNA ligase 4 deficiency SCID	Autosomal recessive
	*PRKDC*	DNA-PKCS deficiency SCID	Autosomal recessive
	*NHEJ1*	Cernunnos-XLF deficiency SCID	Autosomal recessive
	*DCLRE1C*	Artemis SCID	Autosomal recessive

T-B-NK-	*ADA*	Adenosine deaminase deficiency SCID	Autosomal recessive
	*AK2*	Reticular dysgenesis SCID	Autosomal recessive

## Data Availability

The data used to support the findings of this study are available from the corresponding author upon request.
